# The Prognostic Indicators of Progression From Solitary Plasmacytoma to Multiple Myeloma: A Narrative Review

**DOI:** 10.7759/cureus.92844

**Published:** 2025-09-21

**Authors:** Jeremy I Purow, Alejandra Viera, Shaun M Crist, Melissa Ruprich, Stephanie Ocejo, Joanna Herrera, Peter P Wahba, Marco Ruiz-Andia

**Affiliations:** 1 Oncology, Florida International University, Herbert Wertheim College of Medicine, Miami, USA; 2 Malignant Hematology/Bone Marrow Transplant/HIV Oncology, Miami Cancer Institute, Miami, USA

**Keywords:** abnormal plasma cells, biomarkers, cytogenetic abnormalities, multiple myeloma, plasmacytoma

## Abstract

Solitary plasmacytomas (SPs), encompassing both solitary bone plasmacytoma (SBP) and extramedullary plasmacytoma (EMP), are rare plasma cell neoplasms that carry a risk of progression to multiple myeloma (MM). This review synthesizes current literature on the prognostic indicators predictive of such progression. High-risk cytogenetic abnormalities have been consistently linked to faster transition to MM. Other factors, such as tumor size, age, and persistent M-protein levels after treatment, are also prognostic factors. While radiotherapy remains the cornerstone of treatment, its curative potential is influenced by these indicators. Time to progression to MM is highly variable. This review highlights the need for standardized risk stratification systems to guide surveillance and treatment decisions in patients with SP.

## Introduction and background

Solitary plasmacytomas (SP) are rare clonal plasma cell neoplasms characterized by localized proliferation of malignant plasma cells, without the systemic features that define multiple myeloma (MM), such as hypercalcemia, renal insufficiency, anemia, or diffuse osteolytic lesions [1,2]. SP accounts for approximately 5-10% of all plasma cell dyscrasias and can be subdivided into two primary forms: solitary bone plasmacytoma (SBP), which arises within the bone marrow, and extramedullary plasmacytoma (EMP), which originates in soft tissue, most commonly within the upper respiratory tract [3,4]. Less commonly, patients may present with multiple solitary plasmacytomas (MSP), characterized by more than one lesion without systemic myeloma.

The clinical significance of SP lies in its potential to progress to MM, a transition observed in a substantial subset of patients, especially those with SBP. The reported risk of progression varies widely, ranging from 10% to 70%, depending on patient characteristics, biological markers, and length of follow-up [1,5]. While radiation therapy can be curative in some cases, especially for EMP, the majority of patients with SBP eventually relapse or evolve into systemic myeloma, often within the first few years of diagnosis [2,6].

Understanding the prognostic factors associated with this progression is crucial. Traditional risk stratification has relied heavily on clinical features such as age, lesion size, and location, as well as biochemical markers like the presence of serum M-protein and bone marrow plasma cell infiltration [3]. However, recent advancements in molecular biology and cytogenetics have introduced a more nuanced view. Cytogenetic abnormalities such as del(17p), t(4;14), and +1q gain have emerged as powerful predictors of disease behavior in plasmacytoma [2]. Additionally, CXCR4/stromal-derived factor 1 (SDF-1) signaling and other immune-modulatory pathways suggest an evolving molecular understanding of the disease progression [1].

Despite these insights, plasmacytomas remain underrepresented in clinical trials, and the evidence base guiding their management and prognostication is largely limited to retrospective analyses and small cohort studies [6,7]. This has led to heterogeneity in clinical practice and uncertainty regarding optimal treatment strategies, especially in patients with high-risk features. This review aims to synthesize the current literature on prognostic indicators for SP, with a specific focus on the cytogenetic, clinical, and treatment-related factors that influence progression to MM. By identifying and organizing these predictors, we hope to clarify risk stratification approaches and highlight future avenues for research that may aid in earlier intervention and improved patient outcomes.

## Review

Patient demographics 

Patient demographic factors may contribute to prognostic indicators for SP. A study using the SEER database studied over 1600 patients throughout 1998 to 2007 with diagnosed SP to determine survival rates and clinical outcomes as they pertain to gender, ethnicity, and age [8].

Gender Differences

Multiple studies have demonstrated a higher male predominance in patients with SBP at a ratio of 1.5-2.4:1 [8]. In patients with EMP, it was noted that males also have a higher predominance, especially involving the upper respiratory tract at a ratio of 3:1 [4].

However, the SEER-based study found contrasting results with respect to survival, reporting that males had a 64.1% survival rate compared to 53.8% in females [8]. The study did not identify any gender-based biological or clinical factors to explain this discrepancy, suggesting that further research is needed to validate and understand this discrepancy. The low incidence of cases and a lack of large prospective trials are potential confounders.

Racial Differences

African Americans have a notably higher incidence of SP when compared to Caucasians and other ethnicities, a finding that is congruent with other studies [6,7]. Upon analysis of this potential correlation, there were no racially biased survival advantages or disadvantages for SP. It is important to note that this could be due to limited data availability; thus, further studies to look into the component of ethnicity are warranted. 

Age

The incidence of SP increases with age, with the majority of cases reported in individuals over 60 years old. Consequently, survival rates tend to decline with increasing age [8]. One study reported a particularly notable decrease in prognosis and a higher risk of progression to MM among patients over age 60 [5]. However, analysis reveals that several factors may contribute to this trend. For instance, older patients may receive less aggressive treatment in favor of maintaining quality of life and may be less likely to qualify for clinical trials due to comorbid conditions. 

Overall, plasmacytomas have a very limited amount of research, which makes it difficult to ascertain which demographic factors play a role in determining clinical outcome or survival rates. Therefore, this review identifies an area for further research regarding the role of patient demographics in the progression of SP to MM. However, preliminary data suggest that older age may be associated with increased risk of progression to MM.

Clinical predictors and complications that affect progression

In addition to patient demographics, certain clinical features at diagnosis correlate with increased risk of MM progression. Literature demonstrates that tumor size >5 cm, persistent M-protein after treatment, bone marrow involvement, and systemic disease may all be associated with higher rates of progression [3]. 

Bone Marrow Involvement

SPs may or may not involve the bone marrow, impacting their likelihood of progressing to MM. The presence of bone marrow involvement increases the progression risk to MM to 20-60% within the next three years, contrasting with a 10% rate when no bone marrow involvement is observed [9]. Another study concurs with this and reports patients with bone SP progress to MM more frequently than those with extramedullary SP [5]. Additionally, the prognosis worsens significantly in SP of the bone marrow with recurrence or progression to systemic disease [10]. Therefore, once a person is diagnosed with SPs that involve the bone marrow, frequent monitoring is needed to prevent the possibility of extensive bone marrow involvement and progression to MM. 

Tumor Characteristics

Tumor size and location may also play a role in the risk of progression. An older study revealed that after analyzing 46 patients who had SPs, bone presentation and older age were found to be predictive of progression of plasmacytoma to MM and a poorer disease-free survival [11]. The study also found that a tumor size <5 cm improved the local control rate and concluded that anatomic location did not predict outcome. In contrast, alternative studies have found that vertebral involvement was associated with worse local control outcomes, along with older age and larger tumors, and it has been shown that patients who progressed to MM had significantly worse 5-year survival rates than those who did not [5,12]. These clinical diagnostic factors may help to identify those at risk of progression to MM. Therefore, more clinical randomized studies need to occur to assess the impact of anatomic location on progression to MM due to the variability of reports.

Additionally, the high M-protein and the persistence of M-protein after treatment can be an adverse sign of progression [3]. M protein levels may be measured before and after radiation therapy. While it is reported that patients with increased serum M protein before treatment do not correlate with an increased risk, patients with a year-long persistent serum M protein after radiation treatment are associated with a worse prognosis [13]. In addition to the occurrence of MM, there may be the finding of plasmacytomas outside of the bone marrow, similar to EMPs in patients who already have MM. These patients usually have an extremely poor prognosis and require immediate therapy [14].

Complications

Complications of SP are feared due to their association with the systemic complications of MM. Whether originating from MGUS or a plasmacytoma, there is a constant concern about progression to this systemic disease. MM diagnosis relies on the presence of organ damage caused by immunoglobulin infiltration or clonal plasma cells, presenting as hypercalcemia, renal failure, anemia, and osteolytic bone lesions [15]. 

Molecular and cytogenetic predictors of plasmacytomas

While older studies focused on the clinical signs and demographics of SP, newer studies emphasize the role of molecular and cytogenetic predictors of plasmacytomas. It is hypothesized that cytogenetic changes triggered by inhalation of chemicals, overdose of irradiation, viral infection, and disorders in the mononuclear phagocyte system may contribute to the development of plasmacytomas [1]. Recent studies name molecular and cytogenetic abnormalities among the most reliable prognostic indicators of progression from SP to MM. Different genetic variations contribute to the formation and the severity of plasmacytomas. According to the literature, t(4;14) translocation is very frequent among patients with plasmacytomas and can be used as a powerful predictor of disease progression [2].

High-Risk Cytogenetic Abnormalities

A study looking at different markers associated with the severity of SBPs showed that High-Risk cytogenetic abnormalities increased the likelihood of progression to MM [2]. In this study, the electronic medical records of patients diagnosed with plasmacytoma from January 1, 2012, to December 31, 2022, were used. The data obtained included bone marrow plasma cell percentage, the presence of high-risk cytogenetics by FISH, hemoglobin, creatinine, and serum-free light chains. To determine whether the cytogenetics are classified as high-risk or non-high-risk, the Mayo Stratification of Myeloma and Risk-Adapted Therapy (mSMART) guidelines were used. The results of this study demonstrated that del(17p), t(14;16), t(4;14), or +1q (gain or amplification) posed a high risk of transition to MM, especially deletion of 17p and gain 1q abnormalities [2]. The fact that these were classified as “High Risk” means that the presence of the mentioned cytogenetic abnormalities increases the risk of progression from SBP to MM. In summary, these biomarkers were a good predictor of the conversion of plasmacytomas into MM, as patients who had these cytogenetic alterations experienced a short time to progression to MM. Most specifically, in this retrospective cohort study of 114 patients with SBPs, HR biomarkers showed a time to progression to MM of eight months, as compared to those without HR biomarkers, which was 42 months. 

Additionally, cytogenetic changes such as del(1p) and del(14p) in chromosome 13, as well as insertions in 1q and 9q of chromosome 19, are contributors to the formation of plasmacytomas [1]. This is probably because these cytogenic changes interfere with normal cellular processes and may progress to plasmacytomas. For example, del(1p) and del(14p) in chromosome 13 can lead to the loss of tumor suppressor genes, which leads to unregulated cell growth. Additionally, there are molecular factors associated with the development of plasmacytoma at surgical wound sites. In regions of increased trauma and inflammation, there is an increase in the expression of SDF-1, which is an immune cell chemotaxis [1]. After undergoing chemotherapy, the myeloma cells with receptor CXCR-4 attach to the SDF-1, which increases the proliferation of plasma cells and the plasmacytoma formation. In theory, this would lead to a greater risk of progression to MM; however, more research is needed to further determine this link.

One article studied the cytogenetic abnormalities of different patients presenting with EMP [16]. This was accomplished by conducting a FISH on tissue from 41 patients in different institutions with a diagnosis of EMP, the majority located in the upper digestive tract. Some of the cytogenetic aspects observed in these patients were breaks in the 14q32 region and t(4;14)(p16;q32) translocations [16]. In addition, 40% of the patients demonstrated a loss of chromosome region 13q14. Furthermore, 82% of the cases showed chromosomal gains, with duplications of one of the following chromosomes (1, 3, 5, 8, 9, 11, and 15). On the other hand, the translocations t(11;14)(q13;q32), t(14;16)(q32;q23) or t(8;14)(q24;q32) were not observed in these patients [16]. The translocation t(11;14) is common in MM patients, which suggests that it could be strictly associated with bone marrow involvement. From the previously discussed clinical aspects that may increase the risk of progression, bone marrow involvement is linked to a higher risk of progression.

Another study investigated the cytogenetic alterations of patients with extramedullary MM. The extramedullary manifestations studied were extramedullary disease, skeletal extramedullary disease, and solitary EMPs. This was accomplished by combining the FISH technique and cytoplasmic immunoglobulin staining [17]. Some of the locations of the plasmacytomas in these patients were the nose, sphenoidal sinus, and uterus. The results of the study revealed that the patients had a t(4;14), del(17p13), or del(13q14). On the other hand, the patients in the other two groups had del(17p13), del(13q14), and t(4;14) [17]. In addition, patients with plasmacytomas did not reveal MYC-overrepresentation (an oncogenic transcription factor that regulates growth and cell proliferation), as opposed to the other two groups [17]. Therefore, these may be beneficial in identifying those with extramedullary progression of myeloma.

Research on the cytogenetic variations of plasmacytomas is very limited, and only a few studies focus solely on plasmacytomas. Based on the literature search, t(4;14) is very frequent among patients with plasmacytomas.

Time of progression from SP to MM

Determining risk factors, as aforementioned, for the progression of SP to MM is crucial, as around 50% of SBP cases and 15% of EMP cases progress to MM [1]. Various studies assess the length of time it takes for patients with plasmacytoma to develop MM. However, progression from SP to MM is highly variable and unpredictable, occurring in a variable timeline after diagnosis [18,19,20,21]. The discussed variability emphasizes the importance of using other factors, such as clinical and cytogenetic factors, to determine a treatment plan.

In an interesting case from Korea, a patient with a SP progressed to MM in as little as two months [18]. However, studies from across the world show that most patients with plasmacytoma take significantly longer to progress to MM. In a retrospective study, 46 patients with plasmacytoma were followed. 54% of the patients developed MM with a median time of progression of 18 months [19]. Additionally, two studies found that the average time of progression was 21 months. First, a study from Switzerland notes that of 258 patients with plasmacytoma, the median time to development of MM was 21 months [20]. Additionally, in a study from India of 60 patients with SP, the average time of progression from SP to MM was 21 months as well [21]. Similarly, a study conducted on patients from Florida reported that of the 32 patients in the study with plasmacytomas, there was a median time of 25.1 months for patients to progress to MM [22]. 

However, other studies demonstrate a slightly longer period of progression. Specifically, in an older study from Brazil, the average time for patients to progress from plasmacytoma to MM was 41 months with a significant margin of error [23]. On the other hand, a retrospective analysis from Japan of 51 patients with SBP found the median time to progression to MM was 10.5 months, while those with EMP progressed in 18.6 months [24]. Clearly, there is seemingly no uniform timeline for the progression from plasmacytoma to MM. Time of diagnosis may play a role in determining the length of progression as well. Interestingly, one study based on 46 cases of plasmacytoma found that there was little evidence that the risk of progression to MM significantly decreases as time since the diagnosis of plasmacytoma increases [25]. Some patients with plasmacytoma even progressed to MM 17 years after the initial diagnosis of plasmacytoma.

Overall, the median time it takes for patients with plasmacytoma to develop MM significantly varies across different retrospective studies of patients with plasmacytomas. The range of individual patients developing MM from plasmacytoma varies from a matter of months to over 17 years. Therefore, time to progression should not be used in isolation when determining prognosis. Additionally, because of this wide variability, it is essential to identify clinical and molecular markers that can better predict an individual’s risk for MM. The use of this data will help guide the treatment of patients who initially present with plasmacytoma.

Treatment-related outcomes of plasmacytoma

As previously discussed, once SP is diagnosed, the time to progression to MM can be highly variable. Determining the most appropriate course of treatment is therefore critical in preventing progression. However, the literature reveals ongoing controversy regarding optimal management. Identifying patient-specific risk factors may help guide treatment selection, and tailoring therapy for high, intermediate, and low-risk patients could prove beneficial.

Radiation Therapy

Radiation therapy is currently the standard treatment used for plasmacytomas. It provides long-term control in the cases of SBP and has the potential to be curative in EMPs [26]. An exploration completed by the European Expert Panel is in agreement with this, stating that despite the aggressive and locally destructive nature of SPs, they are highly sensitive to radiation and have high response rates to this type of treatment [6]. Relapse rates for those receiving a radiation dose of 40-50 Gy were only 12%, while patients who received no radiotherapy had a relapse rate of 60% [6]. The local response rate to radiotherapy is even higher at 80-90% and was found to be highest in tumors less than 5 cm in diameter [6]. However, a limitation noted in both of these articles is that most studies on the treatment of plasmacytomas have been based on retrospective rather than prospective studies, limiting their control over bias and confounders.

Radiation dose remains a point of debate. A large multi-institutional study found no dose-response relationship beyond 30-35 Gy [26]. However, 40-45 Gy remains standard in practice. This may be due to a retrospective study that reported a local control rate of 94% with ≥40 Gy versus 69% for lower doses [27]. In the absence of phase III trial data, most physicians follow published guidelines from the National Comprehensive Cancer Network and the United Kingdom Myeloma Forum. Higher doses may be required for bulky tumors >5 cm [26]. Evidence has been found, calling for the recommendation of 40-50 Gy over four weeks at 1.8-2.0 Gy per fraction, with the radiation field covering all involved tissues plus at least a 2 cm margin to reduce recurrence risk [6].

Surgical Intervention

Surgical intervention is generally reserved for specific indications, including pathologic fracture or instability of a weight-bearing long bone requiring fixation, or spinal cord compression necessitating decompressive surgery [6,26]. If surgery is required, it is typically completed before radiation therapy, with definitive radiation therapy beginning 4-6 weeks after the post-op period [26]. Definitive surgical excision has only been determined to be acceptable for small tumors with clear margins. In the absence of these specific parameters, surgery alone without radiation therapy leads to a high local recurrence risk [26,6].

Chemotherapy and Novel Agents

The role of chemotherapy is less clear. Some studies found that adjuvant chemotherapy did not affect the incidence of progression to MM, but did delay progression from 39 to 59 months [6]. This trial (which had a median follow-up of 8.9 years) found that 15 out of 28 patients who received radiotherapy alone progressed to myeloma, while only three out of 25 patients in the combined treatment group progressed to myeloma. The survival difference between these two groups was significant (p<0.01), with the combined therapy group having an advantage [7].

Other Factors Affecting Treatment

Cytogenetics may also be used to determine a course of treatment, further indicating their evolving importance in the prognosis of the progression of SP to MM. In one study, 10 out of 19 (52%) of patients who relapsed with prominent hematogenous EMD involvement had high-risk cytogenetics at the time of relapse [28]. Furthermore, the incidence of EMD was significantly increased in patients with high-risk disease at baseline and with baseline cytogenetic abnormalities; this led to the conclusion that hematogenous EMD spread was more prevalent in genomically defined high-risk myeloma [29]. As a result, EMD-unique genes may potentially be identified as targets for new drugs or for different treatment approaches.

The older studies noted a lack of evidence of the benefit of combined modality therapy over radiation therapy alone for patients with SP [6,26]. In a more recent study, the relapse rate was 67% for patients receiving radiotherapy alone and 46% for those receiving the bortezomib-based combined regimen [7]. The patients in this study had low bone marrow plasma cell involvement (<10%) [7]. The overall survival probability for the whole population of the study at five years was 92% and at 10 years was 63%. No significant difference was found in overall survival when comparing the type of treatment (p=0.44). However, this study had some limitations, as it was a retrospective study over 24 years that did not have control over the consistency of diagnostic procedures and had varying availability of drug treatments [7]. One of its strengths, however, was that it was the first study to focus on SBP using recent diagnostic criteria of plasma cell involvement being <10% and the MRI criterion to confirm that the lesion was solitary [7]. The results from this paper show that adjuvant chemotherapy and new drugs, along with the standard treatment of radiotherapy, can improve progression-free survival rates in those with SBPs. However, prospective trials need to be completed to determine the initial staging of SBPs, as this can help determine the initial treatment selected and the monitoring of the plasmacytomas.

In patients with paraskeletal involvement, a proteasome inhibitor regimen followed by stem cell transplantation was found to be the best option. Those who can undergo high-dose therapy may have a similar regimen to those with myeloma or lymphoma and receive bortezomib, thalidomide, and dexamethasone (VTD)-RVD/cisplatin, doxorubicin, cyclophosphamide, and etoposide followed by stem cell transplantation [29]. One study concluded that the use of novel drugs helps to decrease the detrimental effect of paraskeletal involvement and that both proteasome inhibitors and lenalidomide are effective in treatment [29]. However, it is important to note that the data on these newer agents is limited. These studies have small sample sizes, and there has been a lack of controlled trials, making it difficult to make specific recommendations on using these agents.

Overall, the emerging evidence regarding treatment for SP varies, but it points toward the value of incorporating prognostic indicators, such as tumor size, anatomic location, cytogenetic abnormalities, and extramedullary infiltration, when deciding on treatment intensity. Patients with high-risk features may benefit from adding systemic therapy or novel agents up front, but current data are limited to small retrospective studies. Larger, prospective trials that account for these risk factors are needed to better define treatment strategies aimed at delaying or preventing progression to MM.

## Conclusions

Despite being rare plasma cell neoplasms, SPs carry a significant risk of progression to MM. This review synthesizes current literature and highlights marked variability in progression timelines, clinical risk factors, treatment strategies, and outcomes driven by both clinical and cytogenetic heterogeneity. To assess the lack of standardization in the diagnosis, surveillance, and treatment of SP, this review calls for the incorporation of molecular testing (like FISH, as mentioned in the literature) into diagnostic workup. SP patients are vastly underrepresented in prospective clinical trials, and further trials are warranted. While multiple identifiable risk factors for the progression of SP to MM exist, no universally approved guidelines exist to work up these patients. Therefore, the development of a standardized risk surveillance tool that combines cytogenetic, clinical, and demographic markers may help to determine a patient’s risk of developing MM.
